# A survey of perioperative medicine services with a focus on provision for older surgical patients in the UK and Republic of Ireland: SNAP-3

**DOI:** 10.1016/j.bja.2024.12.043

**Published:** 2025-05-19

**Authors:** Claire J. Swarbrick, Amy Donnelly, SNAP-3 collaborators, Karen Williams, Andrea Haren, Bob Evans, Thomas Poulton, Akshay Shah, Judith S.L. Partridge, Iain K. Moppett

**Affiliations:** 1 Anaesthesia and Critical Care, Injury, Recovery and Inflammation Sciences, University of Nottingham, Nottingham, UK; 2 Centre for Research and Improvement, Royal College of Anaesthetists, London, UK; 3 Royal Devon University Healthcare NHS Foundation Trust, Exeter, UK; 4 Vincent's University Hospital, Dublin, Republic of Ireland; 5 Patient, Carer and Public Involvement and Engagement, Royal College of Anaesthetists, London, UK; 6 Department of Anaesthesia, Perioperative Medicine, and Pain Medicine, Peter MacCallum Cancer Centre, Melbourne, VIC, Australia; 7 Department of Critical Care, University of Melbourne, Melbourne, VIC, Australia; 8 Research Department of Targeted Intervention, University College London, London, UK; 9 Nuffield Department of Clinical Neurosciences, University of Oxford, Oxford, UK; 10 Department of Anaesthesia, Hammersmith Hospital, Imperial College Healthcare NHS Trust, London, UK; 11 Perioperative Medicine for Older People Undergoing Surgery (POPS), Guy's and St Thomas' NHS Foundation Trust, London, UK; 12 School of Population Health and Environmental Sciences, Faculty of Life Sciences and Medicine, King's College Hospital, London, UK

**Keywords:** ageing, frailty syndrome, geriatric medicine, health services, manpower, perioperative care, surgery

## Abstract

**Background:**

Perioperative medicine aims to improve care for high-risk patients, and is endorsed by national guidelines in the UK and Republic of Ireland (ROI). However, comprehensive perioperative medicine services are not yet uniformly available. This survey addressed the current state of perioperative medicine services for older surgical patients in the UK and ROI and how these services align with current national guidance.

**Methods:**

A survey was distributed electronically to all publicly administered UK and ROI hospitals performing surgical procedures. The survey examined perioperative care against national recommendations regarding service organisation and conduct.

**Results:**

Of 339 eligible hospitals, 54.9% (186/339) responded. A hospital frailty lead was appointed in 54% (101/186) of hospitals, and 9% (16/186) had a designated anaesthetist for cognitive impairment. Hospital anaesthetic services outside the theatre were focused on preoperative assessment clinics (146/172), with few reporting routine postoperative involvement (17/166). Nurse-led preoperative assessments of frailty, cognition, and delirium risk were conducted in 49.5% (90/182), 44.3% (78/176), and 13.7% (24/175) of hospitals, respectively. The Clinical Frailty Scale was used in 87.0% (147/169) of hospitals for frailty screening. The 4 ‘A's Test (45.7% [85/186]) and Abbreviated Mental Test (43.0% [80/186]) were the preferred cognitive assessment tools.

**Conclusions:**

The survey highlights the variation in perioperative medicine services that exist for older surgical patients despite national guidelines advocating their widespread implementation. Opportunity exists to develop interspecialty perioperative services further and promote identification of frailty, cognitive impairment, and delirium, all of which negatively impact postoperative outcomes for older surgical patients.

**Editor's key points**

• Although endorsed by national guidelines, perioperative medicine services are not widely available.
• This electronic survey, completed in 2023, analysed the perioperative medicine services for older surgical patients in the UK and Ireland, and how they aligned with current national guidance.
• With a response rate of about 55%, the survey identified variation in perioperative medicine services for older surgical patients despite national guidelines advocating widespread implementation.
• This variation in perioperative medicine delivery is attributed to existing interspecialty structures, workforce constraints, and limited resources.
• Opportunities exist for local hospitals to align with the standards of care recommended by guidelines for assessing and managing frailty, cognitive impairment, and postoperative delirium with the aim of improving outcomes for older surgical patients.

The number of older people undergoing surgical procedures is increasing each year; a projected one in five people aged 75 yr or older will undergo surgery annually in England by 2030 with an estimated cost of £2.7 billion.^1^ Age-associated conditions including frailty and multimorbidity predispose patients to postoperative complications and death after surgery.^2^, ^3^, ^4^, ^5^, ^6^, ^7^, ^8^, ^9^

Perioperative medicine is defined as *‘the practice of patient-centred, multidisciplinary, and integrated medical care of patients from the moment of contemplation of surgery until full recovery’*.^10^ Recent reports from the Association of Anaesthetists, Royal College of Anaesthetists (RCoA), British Geriatrics Society (BGS), Centre for Perioperative Care (CPOC), and National Emergency Laparotomy Audit (NELA) all advocate involvement of anaesthetists, geriatricians, and surgeons throughout the perioperative pathway.^11^, ^12^, ^13^, ^14^, ^15^, ^16^ These organisations specifically recommend appointing a named lead anaesthetist for perioperative medicine and cognitive impairment and designating a staff member accountable for the care of older people with frailty during their hospital stay. In addition, they advocate for hospital-wide guidelines on delirium management and implementation of a perioperative care pathway for individuals living with frailty who are undergoing surgery.^11^, ^12^, ^13^, ^14^, ^15^, ^16^ Despite national endorsement of perioperative medicine, and work to identify barriers and enablers to service delivery, whole-pathway multidisciplinary care is not yet universally established throughout the UK National Health Service (NHS) or the Republic of Ireland (ROI).^17^^,^^18^

Previous surveys indicate interest from both anaesthetists and geriatricians in developing perioperative medicine services; however, barriers, including inadequate funding, clinician availability, and training, exist.^17^^,^^18^ In 2016, 65% of UK hospitals conducted high-risk anaesthetic preoperative clinics.^19^ A 2017 survey found that 42% of trusts had geriatricians providing some preoperative assessment services, with proactive postoperative care offered by geriatricians in 48.1% of trusts, particularly in orthopaedics and general surgery.^17^ Surveyed lead anaesthetists for perioperative medicine identified their priorities as shared decision-making, team development, frailty screening and management, and predicting postoperative morbidity. Nonetheless, fewer than a quarter of hospitals had access to specific interventions for managing frailty or cognitive impairment.^19^ Notably, the details of how frailty is screened for in the preoperative setting across a large sample of UK hospitals have not been reported. In addition, although the roles of anaesthetists and geriatricians in perioperative medicine will have evolved since the earlier surveys, there is still a lack of information regarding anaesthetic contribution to the postoperative aspect of perioperative services for older people.

This survey aimed to address the question: What is the current state of perioperative medicine services for older surgical patients (aged ≥ 60 yr) in the UK and ROI, and how do these services align with current national guidance?

## Methods

This cross-sectional, organisational survey constituted one component of the 3rd Sprint National Anaesthesia Project (SNAP-3), also including a cohort study examining frailty, multimorbidity, and delirium in the surgical population,^20^ and a survey assessing the involvement of on-call medical registrars in managing older surgical patients.

The survey was designed to explore how perioperative services are organised and conducted. It was divided into eight sections: hospital details, clinical governance, preoperative assessment organisation, preoperative frailty screening, preoperative cognitive assessment, preoperative assessment of delirium risk, postoperative delirium screening, and postoperative care services. The survey was constructed by an expert panel including anaesthetists, geriatricians, and patient representatives (Supplementary information). The content was derived from national guidelines, with all experts agreeing that every question was essential to ensure content validity.^12^, ^13^, ^14^, ^15^^,^^21^ The survey was designed to minimise missing and inaccurate responses by offering ‘don't know’ or ‘NA (not applicable)’ options where possible and using question filtering to direct respondents to relevant questions only. The survey was piloted by six clinicians for readability, non-ambiguity, and content validity. Ethical approval was not sought because participation was voluntary, no patients were involved, and respondents were not an over-surveyed group.^22^

The survey was distributed by e-mail to the principal investigators (PIs) of the SNAP-3 study based in the UK on the 11th of October 2021. It was promoted through e-mail and social media and at site initiation visits for the SNAP-3 cohort study. The survey could be completed by PIs, who were all consultant anaesthetists, or a delegate, usually a member of the hospital's research department or a resident anaesthetist. The hospital responses from the ROI were coordinated with assistance from the National Clinical Programme for Anaesthesia. The survey was distributed, and data were managed using the REDCap electronic data capture tools hosted at the University of Nottingham.^23^^,^^24^ To reduce the number of hospitals with missing responses, regular reminders were sent to UK sites on 12 occasions from October 2021 until the survey closed in March 2023. Where there were multiple responses from the same hospital, the most recent and complete survey was included in the analysis.

Responses were analysed using descriptive statistics in R (version 4.3.1, R Project for Statistical Computing, Vienna, Austria).^25^ Data are reported as counts of percentages, with median and interquartile ranges (IQRs) used to describe the central tendency and spread. Missing data are recorded in Supplementary Table S1. Where hospitals did not respond to a question, the denominator was adjusted to illustrate the results for those hospitals that did respond. Not all questions were relevant to all hospitals; for example, some hospitals did not have all surgical specialities, and where this was the case, the denominator was adjusted accordingly. Where questions asked for absolute numbers, respondents were asked to refer to the month of September 2021 as a time of relative stability pre-pandemic. Where hospitals were asked to report the screening or assessment tools they used, if a hospital used more than one tool, it could select multiple responses. We defined older patients as those aged ≥60 yr.

## Results

### Survey participation

**Fig 1:**
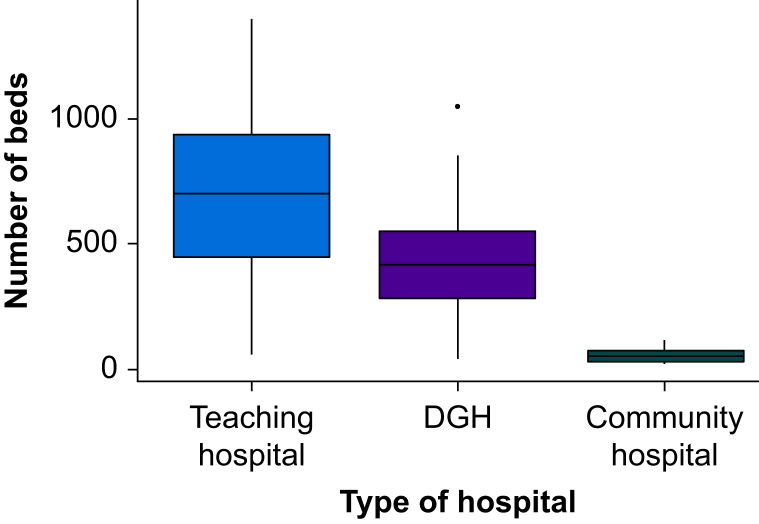
Boxplot of participating hospitals. A boxplot of the types of hospital and number of hospital beds in participating hospitals of the SNAP-3 organisational survey of the UK and Republic of Ireland. DGH, district general hospital.

Fifty-five percent (186/339, 54.9%) of eligible hospitals responded to the survey. Most respondents (119/186, 64%) were from hospitals in England, followed by 23.1% (43/186) from the ROI, 7% (13/186) from Wales, 4.8% (9/186) from Scotland, and 1.1% (2/186) from Northern Ireland. Participation rates varied across regions, with the ROI having the highest participation at 100.0% (46/46), followed by Wales at 72.2% (13/18), England at 51.1% (119/233), Scotland at 30.0% (9/30), and Northern Ireland at 13.3% (2/15). All responding hospitals were publicly administrated NHS- or Health Service Executive (HSE)-funded hospitals. Of the 186 hospitals surveyed, 69.4% (129/186) provided surgical care for patients with hip fracture, 76.9% (143/186) managed patients undergoing elective orthopaedic surgery, and 91.9% (171/186) delivered care for those undergoing general surgery. The responding hospitals encompassed 115 district general hospitals, 61 teaching hospitals, and 10 community hospitals (Fig. 1). Missing data were most prevalent when respondents were asked for absolute numbers, such as when asked for the number of patients reviewed by clinicians and the number of consultant-funded sessions for different roles (Supplementary Table S1).

### Clinical governance

**Fig 2:**
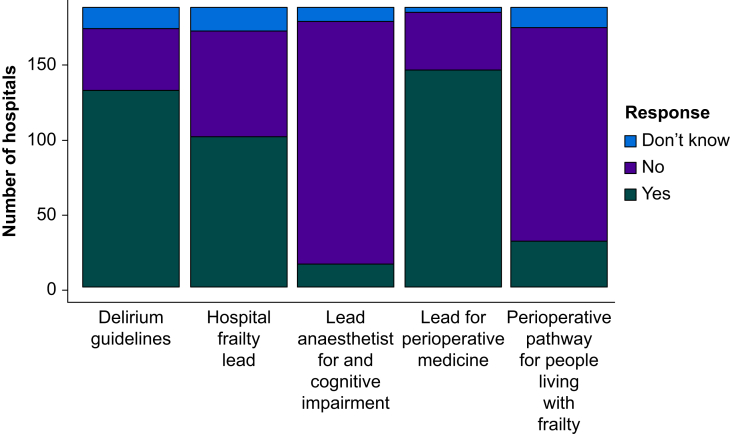
Stacked bar chart of clinical governance measures regarding perioperative medicine. A stacked bar chart illustrating the distribution of clinical governance measures related to the perioperative care of older surgical patients. Data are from the SNAP-3 organisational survey of the UK and Republic of Ireland.

Seventy-nine percent (79.2%, 145/183) of responding hospitals reported having a named anaesthetic lead for perioperative medicine. Nine percent (8.7%, 16/183) reported having a named anaesthetic lead for cognitive impairment. Fifty-nine percent (59.4%, 101/170) reported having a named lead for patients living with frailty. Seventy-six percent (76.7%, 132/172) of hospitals reported guidelines for the prevention and management of delirium, with 18% (31/172) of respondents reporting that their hospital had a perioperative pathway for the management of people living with frailty (Fig. 2).

### Preoperative assessment clinic

During September 2021, there were 80 016 appointments for preoperative assessment of adult patients within the 144 responding hospitals. Of these, 70 472 (88.1%) were anaesthetic nurse-led, 9544 (11.9%) were anaesthetist-led, 275 (0.3%) were led by a geriatrician specialist nurse, and 266 (0.3%) were geriatrician-led (Table 1). The number of clinic appointments provided was recorded in the survey; some patients might have attended more than one appointment to see different clinicians. Eighteen (9.7%) hospitals reported providing a geriatrician-led preoperative assessment clinic. Despite this survey referring to the immediate pre-pandemic period, telephone preoperative assessment was common, with 28% (22 434/80 016) of all consultations undertaken by telephone. Anaesthetists were more likely to see patients in person (8116/9544, 85.0%) than nursing staff (49 466/70 472, 70.2%).

**Table 1:** Preoperative assessment clinics, leading clinicians, and modes of assessment. The number of patients seen in different types of preoperative assessment clinics by clinician leading the clinic and modality of contact. The final column displays the total number of patients reviewed by each clinician's clinic and the overall percentage of patients seen during September 2021 (80 016). IQR, interquartile range.

Preoperative clinic	Face to face		Telephone		Total (%) reviewed face to face or by telephone
	Median (IQR) per hospital	Total for all hospitals	Median (IQR) per hospital	Total for all hospitals	
Nurse-led	233 (78.5–488.5)	49 466	83.0 (10.0–206.0)	21 006	70 472 (88.1)
Anaesthetist-led	29 (10.0–70.0)	8116	0.0 (0.0–5.0)	1428	9544 (11.9)
Geriatrician-led	6 (2.5–10.0)	253	0.0 (0.0–0.0)	13	266 (0.3)
Specialist nurse-led (geriatrics)	5.5 (0.5–10.3)	260	0.0 (0.0–0.0)	15	275 (0.3)

There was variation in the number of anaesthetic consultant-funded sessions (referred to as ‘Programmed Activities’ or PAs in the UK) allocated to preoperative assessment across hospitals. From 172 responding hospitals, the median number of weekly consultant-funded sessions was 5.0 (IQR 2.4–9.0), ranging from 0.0 to 15.0.

### Preoperative assessment of high-risk older patients

Forty-three percent (80/186) of responding hospitals reported *ad hoc* joint consultations (not formal joint clinics) between specialty clinicians with patients present (Supplementary Fig. S1). These interactions were primarily reported between anaesthetists and surgeons (40.0%, 74/186), but also occurred between anaesthetists and geriatricians (16.7%, 31/186) and between geriatricians and surgeons (14.0%, 26/186) in some hospitals. Joint patient clinics were relatively uncommon, with 6.5% (12/186) of hospitals reporting joint clinics between anaesthetists and geriatricians, 3.2% (6/186) between anaesthetists and surgeons, and 1.6% (3/186) between geriatricians and surgeons.

### Preoperative assessment of frailty

In 28.6% (52/182) of responding hospitals, nurse-led preoperative anaesthetic clinics performed frailty screening for all individuals aged 60 yr and above, whereas 18.7% (34/182) of nurse-led clinics screened only selected older patients who were judged to be high risk. Anaesthetist-led preoperative clinics carried out frailty screening for all older patients in 22.2% (38/171) of hospitals and assessed a subset of selected high-risk patients for frailty in 32.2% (55/171) of hospitals. Frailty screening was reported as being performed rarely, inconsistently, or never in 50.5% (92/182) of nurse-led clinics and 48.0% (82/171) of anaesthetist-led clinics among responding hospitals. Four hospitals were unsure whether frailty screening was conducted in nurse-led clinics, and 11 hospitals were uncertain about whether it was conducted in anaesthetist-led clinics (Table 2).

**Table 2:** Preoperative assessment of frailty, cognitive impairment, and the risk of postoperative delirium by different clinicians. Number (%) of the 186 responding hospitals who assess patients for frailty, cognitive impairment, and risk of postoperative delirium in preoperative assessment clinics. The responding hospitals indicated how frequently these assessments were made.

	Clinical setting	All patients ≥60 yr	For selected patients ≥60 yr	Rarely or inconsistently	Never	Don't know or NA
Frailty	Nurse-led clinic	52 (28.0)	38 (20.4)	39 (21.0)	53 (28.5)	4 (2.2)
	Anaesthetist-led clinic	38 (20.4)	55 (29.6)	40 (21.5)	42 (22.6)	11 (5.9)
	Geriatrician-led clinic	13 (7.0)	3 (1.6)	NA	NA	170 (91.4)
Cognitive impairment	Nurse-led clinic	30 (16.1)	48 (25.8)	41 (22.0)	57 (30.6)	10 (5.4)
	Anaesthetist-led clinic	16 (8.6)	54 (29.0)	47 (25.3)	55 (29.6)	14 (7.5)
	Geriatrician-led clinic	10 (5.4)	6 (3.2)	1 (0.5)	NA	169 (90.9)
Risk of postoperative delirium	Nurse-led clinic	10 (5.4)	14 (7.5)	44 (23.7)	107 (57.5)	11 (5.9)
	Anaesthetist-led clinic	6 (3.2)	28 (15.1)	55 (29.6)	84 (45.2)	13 (7.0)
	Geriatrician-led clinic	11 (5.9)	2 (1.1)	1 (0.5)	3 (1.6)	169 (90.9)

**Fig 3:**
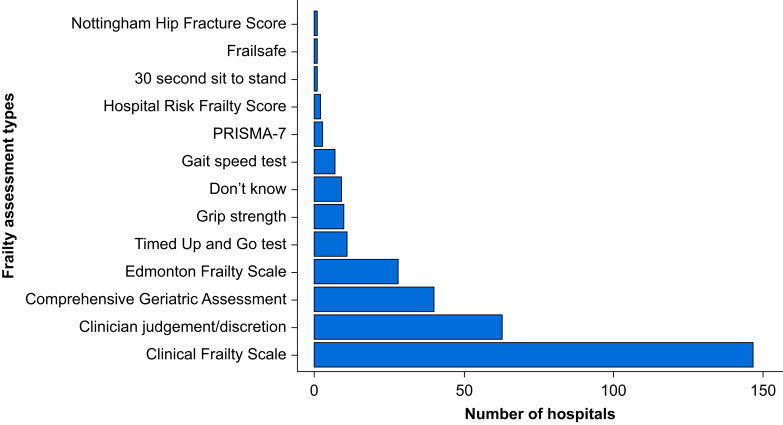
Bar chart of frailty assessment tools used to assess older surgical patients before surgery. Data are from the SNAP-3 organisational survey of the UK and Republic of Ireland. Answers were not mutually exclusive; 26 hospitals did not report which frailty tool they used*.*

The Clinical Frailty Scale (CFS) is the most common method of assessing older surgical patients for frailty, with 87.0% (147/169) of hospitals reporting its use.^26^^,^^27^ Other tools in use included Comprehensive Geriatric Assessment (CGA) (40/169, 23.7%),^28^ Edmonton Frailty Scale (EFS) (28/169, 16.6%),^29^ Timed Up and Go Test (TUG) (11/169, 6.5%),^30^ grip strength (10/169, 5.9%),^31^ gait speed (7/169, 4.1%),^32^ PRISMA-7 (Program of Research on Integration of Services for the Maintenance of Autonomy) (3/169, 1.8%),^33^ 30 second sit to stand (1/169, 1.0%),^34^ Frail Safe (1/169, 1.0%),^35^ and Nottingham Hip Fracture Frailty Score (1/169, 1.0%)^36^ (Fig. 3). No hospitals reported using clinical judgement alone to assess frailty. Nine hospitals reported not knowing which frailty tool they used, and 17 did not answer.

### Preoperative assessment of cognition

Sixteen percent of responding hospitals (16.1%, 30/186) reported that cognition was routinely assessed for all patients aged 60 yr and older in nurse-led preoperative assessment clinics. In 25.8% (48/186) of hospitals, cognition was assessed only for high-risk patients in these clinics. Similarly, in anaesthetist-led preoperative clinics, 8.6% (16/186) of hospitals assessed cognition in all patients aged ≥60 yr, whereas 29.0% (54/186) limited cognitive assessments to high-risk patients. Cognition was rarely, inconsistently, or never assessed in 52.7% (98/186) of responding hospitals' nurse-led clinics and 54.8% (102/186) of responding hospitals' anaesthetist-led clinics. Ten hospitals were unsure whether cognitive assessments were conducted in nurse-led clinics, and 14 hospitals did not know if such assessments were conducted in anaesthetist-led clinics (Table 2).

**Fig 4:**
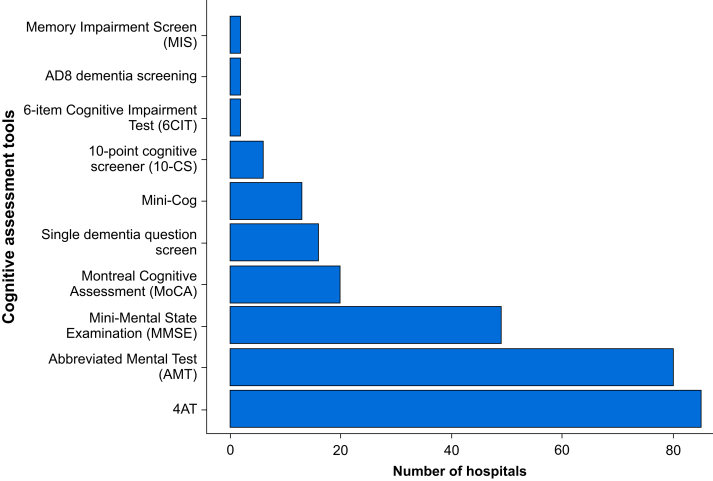
Bar chart showing cognition assessment tools used in older surgical patients. Data are from the SNAP-3 organisational survey of the UK and Republic of Ireland. Answers were not mutually exclusive; 32 hospitals did not report which cognitive assessment tool they used.

The two most frequently used tools to assess cognition in a preoperative setting are the 4 ‘A's Test (4AT) assessment and the Abbreviated Mental Test (AMT), used in 45.7% (85/186) and 43.0% (80/186), respectively (Fig. 4). Twenty-eight hospitals did not answer, and four did not know which tools were used.

### Preoperative risk assessment for postoperative delirium

Fifty-eight percent of responding hospitals (107/186) indicated that nurse-led preoperative clinics never assessed the risk of postoperative delirium. Similarly, 45.2% (84/186) reported that anaesthetist-led preoperative clinics did not assess this risk. In 12.9% of responding hospitals (24/186), nurse-led preoperative clinics specifically assessed the risk of postoperative delirium in all patients aged 60 yr and over or in selected high-risk patients. Anaesthetic-led clinics assessed the risk of delirium in 18.3% of hospitals (34/186), either for all older patients or those deemed high risk. Eleven hospitals were unsure whether the risk of delirium was assessed for in nurse-led clinics, and 13 hospitals did not know if such assessments were conducted in anaesthetist-led clinics (Table 2).

Clinical judgement was the most commonly reported approach in determining if a patient is at risk of developing postoperative delirium (109/186 58.6%). There was no consensus on which tool should be used to formalise this process; 26 hospitals produced their own locally adapted risk assessment, six used the ACS NSQIP,^37^ two used the DEAR tool,^38^ one used the DELPHI tool,^39^ nine did not know, and 33 did not answer.

### Postoperative assessment for delirium

**Fig 5:**
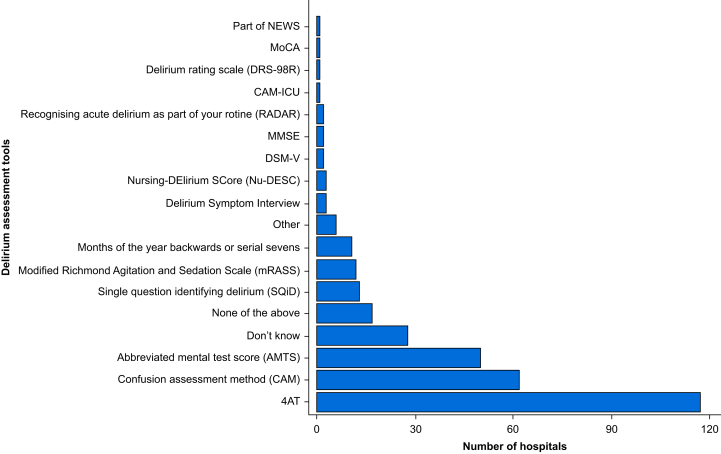
Bar chart of delirium assessment tools used to assess older surgical patients after surgery. Data are from the SNAP-3 organisational survey of the UK and Republic of Ireland. Answers were not mutually exclusive; 28 hospitals did not report which delirium assessment tool they used*.* Montreal Cognitive Assessment (MoCA), Mini-Mental State Examination (MMSE), Diagnostic and Statistical Manual of Mental Disorders, 5th Edition DSM-V, Modified Richmond Agitation and Sedation Scale (mRASS)

The tool most commonly used to detect postoperative delirium was the 4AT assessment (117/186, 62.9%), followed by the Confusion Assessment Method (CAM)^40^ (62/186, 33.3%) and AMT^41^ (50/186, 26.9%) (Fig. 5). Almost a quarter of hospitals (44/186, 23.7%) indicated that they used three or more different tools to detect delirium, whereas 28 responding hospitals (15.1%) did not know how delirium was assessed at their site.

### Postoperative care

The routine provision of postoperative care by consultant anaesthetists is generally limited to postanaesthesia care units (PACUs), enhanced care units, critical care, pain services, and *ad hoc* ward visits. Most responding hospitals (90.0%, 149/166) reported no funded consultant anaesthetist sessions for postoperative care. A small percentage (6.6%, 11/166) had 0.5–4.9 weekly funded sessions, and 3.6% (6/166) had 5–15 weekly sessions. Twenty hospitals were not able to answer how many weekly sessions were funded for postoperative anaesthetic consultant-led care.

Of the 141 hospitals who answered, 84 hospitals reported no weekly funded geriatrician consultant sessions for postoperative care. Almost 20% (19.1%, 27/141) had 0.5–4.9 weekly funded sessions, and 21.3% (30/141) had 5–15 weekly sessions. Forty-five hospitals were not able to answer how many weekly sessions were funded for postoperative geriatrician consultant-led care.

For elective orthopaedic surgery, 11.9% (17/143) had scheduled proactive geriatrician input (Supplementary Fig. S2). In general surgery, 14.6% (25/171) provided proactive geriatrician input for older patients requiring emergency laparotomy, and 9.4% (16/171) for elective surgery (Supplementary Figs. S3 and S4). A reactive, referral-based geriatrician model was more common, available in 55.0% (94/171) of hospitals for emergency laparotomy and 70.8% (121/171) elective general surgery.

Postoperative ward-based multidisciplinary team (MDT) meetings for rehabilitation goal setting and discharge planning are uncommon among older high-risk patients, except for those undergoing surgery for hip fracture. Amongst hospitals treating those with hip fractures, 35.7% (46/129) reported involvement of allied health professionals, 19.4% (25/129) included nurse specialists, 17.8% (23/129) had geriatricians, 7.0% (9/129) had anaesthetists, and 4.7% (6/129) included non-geriatrician physicians (Supplementary Fig. S5). Responses to this survey question were not mutually exclusive, allowing hospitals to report multiple types of professional involvement. MDTs were less frequent for emergency laparotomy and elective surgeries, with fewer than 8% (15/186) of hospitals reporting them for these groups.

## Discussion

This large survey of UK and ROI hospitals describes how perioperative medicine services are currently organised and compares its findings to national guidance. It highlights that perioperative service provision in the UK and ROI continues to fall short of national recommendations with variation noted between centres. There was substantial variation in the governance roles and guidelines supporting perioperative care for older patients across responding hospitals. The national recommendations we used as markers of good practice align with guidelines from the American College of Surgeons (ACS) National Surgical Quality Improvement Program (NSQIP) and the American Geriatrics Society (AGS) Best Practice Guidelines. These guidelines recommend preoperative screening for all surgical patients aged ≥65 yr for frailty, cognitive impairment, and delirium risk. Although the suggested tools differ across nations, the underlying principles are similar. Both the ACS NSQIP/AGS and UK-based guidance emphasise the involvement of geriatricians, physicians, and allied healthcare professionals throughout the surgical pathway.

### Whole-pathway interspecialty perioperative medicine

The RCoA and CPOC share a vision for perioperative medicine encompassing interspecialty involvement from the contemplation of surgery until full recovery.^10^^,^^12^ Anaesthetist's involvement is skewed towards preoperative and intraoperative care. Although anaesthetic consultant involvement in preoperative assessment clinic is ubiquitous, not all UK or ROI consultant anaesthetists are involved in preoperative assessment clinics. In our survey, it was rare for hospitals to report routine postoperative funded sessions for anaesthetists outside pain medicine, enhanced, and critical care.

Geriatrician involvement in the surgical pathway varies markedly across hospitals, with a focus primarily on postoperative care. It appears that the availability of dedicated geriatrician-led preoperative clinics has seen little growth, from 14 trusts in 2017 to 18 hospitals in 2021/22.^17^ However, the increase in *ad hoc* interspecialty patient consultations suggests growing enthusiasm and recognition of the value of timely shared decision-making. The limited presence of scheduled interspecialty clinics and dedicated geriatrician-led preoperative services could indicate gaps in clinical demand, clinician engagement, lack of high-quality supporting evidence, or infrastructure. In addition, the COVID-19 pandemic may have hindered expansion. Barriers to anaesthetic involvement in perioperative care—such as workforce shortages, lack of training, and confidence in meeting care standards—have also been identified as limiting factors in expanding anaesthetic-led services.^18^

Geriatricians are skilled in the management of older people, with expertise in the identification and management of frailty, cognitive impairment, and delirium. Expanding their role into perioperative care benefits patient and hospital level outcomes; however, their already overstretched workforce means that hospitals must find innovative ways to benefit from their experience. Interspecialty clinics or ward rounds, where anaesthetists, geriatricians, nurses, and surgeons can mutually benefit from each other's different experiences, are desirable but staff intensive. Joint working groups to develop guidelines, services, and patient pathways, and perioperative MDTs with a geriatrician attending, can all offer a more efficient means of expanding their clinical reach and educating non-geriatricians.

### Identification of geriatric syndromes in the preoperative setting

Our survey shows an increasing awareness of the importance of screening for frailty, with about half of responding hospitals now assessing frailty in their preoperative assessment clinic. This represents improvement from a previous survey by Bougeard and colleagues^19^ from 2016, where only a quarter of responding hospital perioperative leads reported assessing for frailty before surgery. In contrast, around a quarter of responding hospitals reported never screening for frailty. This suggests a significant disparity in frailty assessment practices among hospitals, although notably this survey was undertaken just as the CPOC–BGS Guideline for Perioperative Care for People Living with Frailty Undergoing Elective and Emergency Surgery was published. This guideline specifies who should be screened for frailty and which tool to choose, so with the implementation of the guideline into practice, a greater number of hospitals might now be undertaking routine frailty screening in the perioperative setting.^14^

Cognitive impairment screening is often perceived as being impractical for a high-turnover preoperative environment.^19^ There appears to have been little increase in the numbers of responding hospitals assessing cognition since 2016; however, the most commonly used screening tool has changed from the mini mental test score (also known as Mini-Mental State Examination) to the 4AT and AMT.^19^ Around a third of hospitals report never assessing patient cognition in the preoperative assessment clinic. The criteria for cognitive screening in responding hospitals shows considerable variation, despite previous guidance encouraging cognitive assessment for any patient with concerns about or signs of mild cognitive impairment or dementia.^15^ Cognitive impairment is a strong predictor of postoperative delirium and impacts perioperative care needs. These include tailored communication, careful medication management, appropriate postoperative staffing, thorough discharge planning, and special considerations for obtaining consent. To make these important modifications to the standard surgical pathway, cognitive impairment should be screened for routinely. The current variations in practice and unfamiliarity with cognitive screening highlights a training gap for anaesthetists and preoperative nursing staff in the use of cognitive screening tools.^18^

Preoperative assessment clinics are generally focused on identifying factors that can be optimised before surgery. However, they may not be well placed for shared decision-making, partly because of the relatively unusual colocation of all relevant parties, and because of their chronology, generally when the patient is well along the surgical pathway. In older patients and those living with frailty and multimorbidity, evidence increasingly supports the role of CGA and optimisation clinics earlier in the preoperative phase of the surgical pathway to support shared decision-making.^42^

As perioperative medicine leads and contributors to preoperative clinics, anaesthetists play a pivotal role in organising and delivering preoperative care. Greater local uptake of national recommendations, including the appointment of a lead consultant for cognitive impairment or the adoption of a perioperative pathway for patients living with frailty, will allow enhanced promotion of the need to screen for geriatric syndromes, such as frailty and cognitive impairment. Raising awareness of geriatric syndromes through guideline implementation alongside consistent screening early in the perioperative pathway results in better detection and optimisation of these issues and informs shared decision-making.

### Delirium risk assessment and postoperative detection

Discussing the risk of postoperative delirium is essential for informed consent and shared decision-making, a priority previously highlighted by a survey of perioperative medicine leads in 2017.^19^ Comprehensive shared decision-making, with discussion of likely postoperative complications and quality of life reduces the likelihood of decisional regret, which occurs in about on one in seven surgical patients.^43^ However, only about half of anaesthetist- and nurse-led clinics preoperatively assess this risk, with most not routinely forecasting the likelihood of this postoperative complication. Moreover, few hospitals use a standard tool to assess the postoperative risk of delirium likely because there is no widely accepted tool for this purpose. Regardless of the method used, clinical judgement, or a standardised tool, assessing delirium risk is vital to ensure appropriate interventions are implemented to mitigate potential postoperative complications and to adequately inform preoperative shared decision-making.^44^^,^^45^

The 4AT was the most commonly used tool to detect postoperative delirium and is used twice as frequently as the CAM. However, about a quarter of responding hospitals reported using three or more tools, and about one in seven were unsure which tool their hospital used. There is a need for broader promotion of simple delirium detection tools in surgical areas. Notably, 71% (20/28) of hospitals that were unsure of the tool used also reported having hospital-wide delirium guidelines, raising concerns about the implementation and effectiveness of these guidelines in perioperative areas.

### Recommendations for frailty, cognitive impairment, and delirium risk assessment

The 2021 CPOC-BGS Guideline for Perioperative Care for People Living with Frailty Undergoing Elective and Emergency Surgery is expected to enhance screening for frailty and cognitive impairment. It advises that all surgical patients aged 65 yr or older be assessed for frailty using the CFS. Patients with a CFS score of ≥5 should also undergo cognitive assessment using tools such as the 4AT, mini-Cog, or MoCA, in addition to a CGA.^14^ In addition, the guideline emphasises assessing patients living with frailty (CFS ≥5) for delirium risk by considering both predisposing and precipitating factors.^14^ This clear guidance should help reduce inconsistencies in the provision and method of screening for geriatric syndromes. Although there are established tools for frailty, cognitive impairment, and delirium, there remains an opportunity to develop a sensitive, specific, and easily implementable tool to forecast risk of postoperative delirium in a preoperative setting.

### Strengths and limitations

This survey included a large sample of hospitals in the UK and ROI to characterise perioperative medicine services. Fifty-five percent of eligible hospitals responded, demonstrating a good response rate. This survey collected information that is not centrally recorded but is of use to policymakers and managers of local and national healthcare services. In combination with the CPOC–BGS guidance, its results can help organisations implement standards of care such as the use of CFS for frailty screening. Survey respondents exhibited limited awareness of geriatric medicine involvement in perioperative services. This lack of awareness could stem from a true low availability of geriatrician input within the hospital or a general unawareness of available geriatrician-led services among those completing the survey. The ROI uses different national guidelines from the UK, accounting for potential differences in service development; however, the literature base is applicable across healthcare systems. The survey closed in 2023; since then, perioperative medicine services have evolved and are likely to be more widespread.

As with most surveys, selection bias was introduced because of hospitals self-selecting to participate. We attempted to minimise this by making survey completion part of the wider SNAP-3 project setup; however, this was not always successful. The survey was unable to recruit all hospitals across the UK and ROI, which contributed to potential selection bias arising from differing response rates between countries. In the ROI, the survey's completion rate benefited from coordination and support provided by the National Clinical Programme for Anaesthesia, which helped facilitate participation. Hospital engagement with surveys and research projects tends to vary depending on local enthusiasm and the level of practical support and leadership within the hospitals and research networks. In addition, the survey contains incomplete data, which are likely missing because of the nature of the questions and the ability of the individuals completing the survey to find the answers. For questions where absolute numbers were requested, there was between 22.6% and 62.4% missingness, calling into question how generalisable these results are. Where responses were missing to questions asking which screening tool is used, a non-response might indicate a lack of familiarity with screening for geriatric syndromes (Supplementary Table S1).

### Conclusions

Perioperative medicine services continue to evolve, yet challenges persist in establishing standardised care across the UK and ROI. The observed variation in perioperative medicine delivery is attributed to existing interspecialty structures, workforce constraints, and limited resources. Variations in practice present an opportunity for local hospitals to align with the standards of care recommended by the CPOC–BGS guideline for assessing and managing frailty, cognitive impairment, and postoperative delirium with the aim of improving outcomes for older surgical patients.

## Authors’ contributions

Initiated the collaborative project, is the guarantor and the grant holder, and revised the draft paper: IKM

Implemented the study in the UK, designed the data collection tools, monitored data collection for the study, cleaned and analysed the data, and drafted and revised the manuscript: CS

Provided expertise in geriatric medicine, designed data collection tools and protocol, and revised the draft manuscript: JP

Coordinated survey activity and data collection in the Republic of Ireland: AD, AH

Designed data collection tools and revised the draft manuscript: TP

Coordinated site and UK-wide activity: KW

Provided expertise in anaesthetics and perioperative medicine, designed data collection tools and protocol, and revised the manuscript: AS

Provided insights from a patient and public perspective and helped to design the patient facing documents: BE

Reviewed the final draft manuscript and contributed to revisions: all authors

## Funding

The Frances and Augustus Newman Foundation and the Royal College of Anaesthetists. They had no input into the design, conduct, or analysis of the study.

## Declaration of interest

IKM is the Director of the Centre for Research and Improvement at the Royal College of Anaesthetists.

## References

[1] Fowler A.J., Abbott T.E.F., Prowle J., Pearse R.M. (2019). Age of patients undergoing surgery. Br J Surg.

[2] Lin H.-S., Watts J.N., Peel N.M., Hubbard R.E. (2016). Frailty and post-operative outcomes in older surgical patients: a systematic review. BMC Geriatr.

[3] Hewitt J., Carter B., McCarthy K. (2019). Frailty predicts mortality in all emergency surgical admissions regardless of age. An observational study. Age Ageing.

[4] Parmar K.L., Law J., Carter B. (2021). Frailty in older patients undergoing emergency laparotomy: results from the UK Observational Emergency Laparotomy and Frailty (ELF) study. Ann Surg.

[5] Sandini M., Pinotti E., Persico I., Picone D., Bellelli G., Gianotti L. (2017). Systematic review and meta-analysis of frailty as a predictor of morbidity and mortality after major abdominal surgery. BJS Open.

[6] Hewitt J., Long S., Carter B., Bach S., McCarthy K., Clegg A. (2018). The prevalence of frailty and its association with clinical outcomes in general surgery: a systematic review and meta-analysis. Age Ageing.

[7] NELA Project Team (2023).

[8] Wolff J.L., Starfield B., Anderson G. (2002). Prevalence, expenditures, and complications of multiple chronic conditions in the elderly. Arch Intern Med.

[9] Roche J.J.W., Wenn R.T., Sahota O., Moran C.G. (2005). Effect of comorbidities and postoperative complications on mortality after hip fracture in elderly people: prospective observational cohort study. BMJ.

[10] Centre for Perioperative Care. What is perioperative care? Available from: https://cpoc.org.uk/about-cpoc/what-perioperative-care#:∼:text=Perioperative%20care%2C%20also%20referred%20to%20as%20perioperative%20medicine%2C,moment%20of%20contemplation%20of%20surgery%20until%20full%20recovery (accessed 4 January 2024).

[11] Royal College of Anaesthetists (2019).

[12] Mythen M.G., Berry C., Drake S. (2015).

[13] National Institute for Health and Care Excellence (2023).

[14] Perioperative Care of People Living with Frailty (2021).

[15] White S., Griffiths R., Baxter M. (2019). Guidelines for the peri-operative care of people with dementia. Anaesthesia.

[16] Royal College of Anaesthetists (2021).

[17] Joughin A.L., Partridge J.S.L., O'Halloran T., Dhesi J.K. (2019). Where are we now in perioperative medicine? Results from a repeated UK survey of geriatric medicine delivered services for older people. Age Ageing.

[18] Partridge J.S.L., Rogerson A., Joughin A.L. (2020). The emerging specialty of perioperative medicine: a UK survey of the attitudes and behaviours of anaesthetists. Perioper Med.

[19] Bougeard A.M., Brent A., Swart M., Snowden C. (2017). A survey of UK peri-operative medicine: pre-operative care. Anaesthesia.

[20] Swarbrick C., Poulton T., Martin P., Partridge J., Moppett I.K. (2023). Study protocol for a national observational cohort investigating frailty, delirium and multimorbidity in older surgical patients: the third Sprint National Anaesthesia Project (SNAP 3). BMJ Open.

[21] (2021). Preoperative assessment and optimisation for adult surgery including consideration of COVID-19 and its implications.

[22] Health Resarch Authority (2017). HRA decision tool. https://www.hra-decisiontools.org.uk/research/.

[23] Harris P., Taylor R., Minor B. (2019). The REDCap consortium: building an international community of software partners. J Biomed Inform.

[24] Harris P., Taylor R., Thielke R., Payne J., Gonzalez N., Conde J. (2009). Research electronic data capture (REDCap)—a metadata-driven methodology and workflow process for providing translational research informatics support. J Biomed Inform.

[25] R Core Team (2023).

[26] Rockwood K., Song X., MacKnight C. (2005). A global clinical measure of fitness and frailty in elderly people. CMAJ.

[27] Pulok M.H., Theou O., van der Valk A.M., Rockwood K. (2020). The role of illness acuity on the association between frailty and mortality in emergency department patients referred to internal medicine. Age Ageing.

[28] Harari D., Hopper A., Jugdeep Dhesi J., Babic-Illman G., Lockwood L., Martin F. (2007). Proactive care of older people undergoing surgery (‘POPS’): designing, embedding, evaluating and funding a comprehensive geriatric assessment service for older elective surgical patients. Age Ageing.

[29] Rolfson D.B., Majumdar S.R., Tsuyuki R.T., Tahir A., Rockwood K. (2006). Validity and reliability of the Edmonton frail scale. Age Ageing.

[30] Podsiadlo D., Richardson S. (1991). The timed “Up & Go”: a test of basic functional mobility for frail elderly persons. J Am Geriatr Soc.

[31] Bohannon R.W. (2008). Hand-grip dynamometry predicts future outcomes in aging adults. J Geriatr Phys Ther.

[32] Peel N.M., Kuys S.S., Klein K. (2012). Gait speed as a measure in geriatric assessment in clinical settings: a systematic review. J Gerontol A Biol Sci Med Sci.

[33] Hoffmann S., Wiben A., Kruse M., Jacobsen K.K., Lembeck M.A., Holm E.A. (2020). Predictive validity of PRISMA-7 as a screening instrument for frailty in a hospital setting. BMJ Open.

[34] Bohannon R.W., Bubela D.J., Magasi S.R., Wang Y.-C., Gershon R.C. (2010). Sit-to-stand test: performance and determinants across the age-span. Isokinet Exerc Sci.

[35] Papoutsi C., Poots A., Clements J., Wyrko Z., Offord N., Reed J.E. (2018). Improving patient safety for older people in acute admissions: implementation of the Frailsafe checklist in 12 hospitals across the UK. Age Ageing.

[36] Wiles M.D., Moran C.G., Sahota O., Moppett I.K. (2011). Nottingham Hip Fracture Score as a predictor of one year mortality in patients undergoing surgical repair of fractured neck of femur. Br J Anaesth.

[37] Berian J.R., Zhou L., Russell M.M. (2018). Postoperative delirium as a target for surgical quality improvement. Ann Surg.

[38] Meehan A.J., Gabra J.N., Distelhorst K.S., Whyde C., Mangira C. (2023). Development and validation of a delirium risk prediction model using a modified version of the Delirium Elderly at Risk (mDEAR) screen in hospitalized patients aged 65 and older: a medical record review. Geriatr Nurs.

[39] Kim M.Y., Park U.J., Kim H.T., Cho W.H. (2016). DELirium prediction based on hospital information (delphi) in general surgery patients. Medicine (Baltimore).

[40] Shi Q., Warren L., Saposnik G., Macdermid J.C. (2013). Confusion assessment method: a systematic review and meta-analysis of diagnostic accuracy. Neuropsychiatr Dis Treat.

[41] Hodkinson H.M. (1972). Evaluation of a mental test score for assessment of mental impairment in the elderly. Age Ageing.

[42] Scarfield P., Sharkey A.R., Dhesi J.K., Modarai B., Tyrrell M.R., Partridge J.S.L. (2024). Preoperative clinical characteristics and 12-month outcomes following operative or non-operative management of asymptomatic aortic aneurysms. Age Ageing.

[43] Wilson A., Ronnekleiv-Kelly S.M., Pawlik T.M. (2017). Regret in surgical decision making: a systematic review of patient and physician perspectives. World J Surg.

[44] Moppett I. (2024). Postoperative delirium: more risk scores or more action?. Age Ageing.

[45] Penfold R.S., Squires C., Angus A. (2024). Delirium detection tools show varying completion rates and positive score rates when used at scale in routine practice in general hospital settings: a systematic review. J Am Geriatr Soc.

