# Supplementary material for: A survey of perioperative medicine services with a focus on provision for older surgical patients in the UK and Republic of Ireland: SNAP-3
Source: Br J Anaesth. 2025 May 19;135(1):155–65. doi: 10.1016/j.bja.2024.12.043 (PMC12226739; doi:10.1016/j.bja.2024.12.043)
Supplement: Multimedia component 3 [file mmc3.pdf]

SNAP 3: Organisational Survey (S2)

We are writing to invite you to complete this short online survey which aims to describe the perioperative management of older patients undergoing surgery. We the hope the findings of the survey will help us understand variation in perioperative care across the country.

The survey includes questions on how perioperative medicine services are organised at your hospital site, including information regarding services delivered in September 2021. The survey refers to adult services only.

It is unlikely that you'll be able to complete the whole survey in the same sitting. If you are not able to provide the information requested, we are grateful to you for forwarding the survey to someone else in your institution to complete. The survey can be passed between colleagues to complete relevent sections of the survey and can be saved and returned to by pressing 'Save & Return'. You may need to contact colleagues from perioperative medicine, geriatric medicine, preoperative assessment services, surgery and administration staff.

For the purposes of this survey:
• 'Older' refers to patients who are aged 60 years old and over.
• 'Elective surgery' refers to interventions that are planned or booked in advance of routine admission into hospital. Timing to suit patient, hospital and staff.
• 'Emergency surgery' refers to NCEPOD classified immediate, urgent and expedited interventions where the surgery is required within days, hours or minutes.

References
1. Royal College of Anaesthetists. Chapter 2: Guidelines for the Provision of Anaesthesia Services for the Perioperative Care of Elective and Urgent Care Patients. 2021
2. Royal College of Anaesthetists. Perioperative Medicine Programme http://www.rcoa.ac.uk/perioperativemedicine, 2019
3. Getting it Right First Time Programme National Speciality Report. Geriatric Medicine. https://gettingitrightfirsttime.co.uk

There are 13 pages of questions in this survey. Thank you for taking the time to complete one survey for each hospital within your trust.

General

1a. Which country is your hospital based in?

◯ England
◯ Northern Ireland
◯ Scotland
◯ Wales

1b. Which English hospital site are you completing this form for?

◯ Not listed
◯ Addenbrooke's Hospital
◯ Airedale General Hospital
◯ Alexandra Hospital Redditch
◯ Arrowe Park Hospital
◯ Ashford Hospital
◯ Barnet General Hospital
◯ Barnsley Hospital
◯ Basildon University Hospital
◯ Basingstoke and North Hampshire Hospital
◯ Bedford Hospital
◯ Birmingham City Hospital
◯ Birmingham Women's Hospital
◯ Blackpool Victoria Hospital
◯ Bradford Royal Infirmary
◯ Bridlington and District Hospital
◯ Bristol Royal Infirmary
◯ Broadgreen Hospital
◯ Broomfield Hospital
◯ Burnley General Teaching Hospital
◯ Calderdale Royal Hospital
◯ Charing Cross Hospital
◯ Chase Farm Hospital
◯ Chelsea and Westminster Hospital
◯ Cheltenham General Hospital
◯ Cheshire and Mersey Treatment Centre (CMTC)
◯ Chesterfield Royal Hospital
◯ Churchill Hospital
◯ Clatterbridge Hospital
◯ Colchester General Hospital
◯ Conquest Hospital
◯ Countess of Chester Hospital
◯ Croydon University Hospital
◯ Cumberland Infirmary
◯ Darent Valley Hospital
◯ Darlington Memorial Hospital
◯ Derriford Hospital
◯ Diana Princess of Wales Hospital
◯ Doncaster Royal Infirmary
◯ Dorset County Hospital
◯ East Surrey Hospital
◯ Eastbourne District General Hospital
◯ Epsom General Hospital
◯ Essex Cardiothoracic Centre
◯ Fairfield General Hospital
◯ Freeman Hospital
◯ Frimley Park Hospital
◯ Furness General Hospital
◯ Glenfield Hospital
◯ Gloucestershire Royal Hospital
◯ Good Hope Hospital
◯ Great Western Hospital
◯ Guy's Hospital
◯ Halton General Hospital
◯ Hammersmith Hospital
◯ Harrogate District Hospital
◯ Heartlands Hospital
◯ Hexham General Hospital
◯ Hillingdon Hospital
◯ Hinchingbrooke Hospital
◯ Homerton University Hospital
◯ Huddersfield Royal Infirmary
◯ Hull Royal Infirmary
◯ Ipswich Hospital
◯ James Paget University Hospital
◯ John Radcliffe Hospital
◯ Kent and Canterbury Hospital
◯ Kettering General Hospital
◯ King George Hospital

◯ King's College Hospital
◯ Kingston Hospital
◯ Leeds General Hospital
◯ Leighton Hospital
◯ Lincoln County Hospital
◯ Lister Hospital
◯ Liverpool Women's Hospital
◯ Luton and Dunstable Hospital
◯ Manchester Royal Infirmary
◯ Medway Maritime Hospital
◯ Milton Keynes University Hospital
◯ Musgrove Park Hospital
◯ National Hospital for Neurology and Neurosurgery
◯ New Cross Hospital
◯ Newham University Hospital
◯ Norfolk and Norwich University Hospital
◯ North Devon District Hospital
◯ North Manchester General Hospital
◯ North Middlesex University Hospital
◯ North Tyneside General Hospital
◯ Northampton General Hospital
◯ Northern General Hospital
◯ Northumbria Specialist Emergency Care Hospital
◯ Nottingham City Hospital
◯ Nuffield Orthopaedic Centre
◯ Ormskirk District General Hospital
◯ Orpington Hospital
◯ Papworth Hospital
◯ Peterborough City Hospital
◯ Pilgrim Hospital
◯ Pinderfields Hospital
◯ Poole Hospital
◯ Princess Alexandra Hospital
◯ Princess Anne Hospital
◯ Princess Royal Haywards Heath
◯ Princess Royal Hospital
◯ Princess Royal University Hospital
◯ Queen Alexandra Hospital
◯ Queen Charlotte's and Chelsea Hospital
◯ Queen Elizabeth Hospital
◯ Queen Elizabeth Hospital
◯ Queen Elizabeth Hospital
◯ Queen Elizabeth Hospital
◯ Queen Elizabeth The Queen Mother Hospital
◯ Queen Victoria Hospital (East Grinstead)
◯ Queen's Hospital
◯ Queen's Hospital
◯ Queen's Medical Centre
◯ Robert Jones and Agnes Hunt Orthopaedic Hospital
◯ Rotherham Hospital
◯ Royal Albert Edward Infirmary
◯ Royal Berkshire Hospital
◯ Royal Blackburn Teaching Hospital
◯ Royal Bolton Hospital
◯ Royal Bournemouth Hospital
◯ Royal Derby Hospital
◯ Royal Devon and Exeter Hospital
◯ Royal Free Hospital
◯ Royal Hallamshire Hospital
◯ Royal Hampshire County Hospital
◯ Royal Lancaster Infirmary
◯ Royal National Orthopaedic Hospital
◯ Royal National Throat, Nose and Ear Hospital
◯ Royal Oldham Hospital
◯ Royal Orthopaedic Hospital
◯ Royal Preston Hospital
◯ Royal Shrewsbury Hospital
◯ Royal Stoke University Hospital
◯ Royal Surrey County Hospital
◯ Royal Sussex County Hospital
◯ Royal United Hospital

◯ Royal Victoria Infirmary
◯ Russells Hall Hospital
◯ Saint Mary's Hospital
◯ Salford Royal Hospital
◯ Salisbury District Hospital
◯ Sandwell General Hospital
◯ Scarborough Hospital
◯ Scunthorpe General Hospital
◯ Solihull Hospital
◯ South Tyneside District Hospital
◯ Southampton General Hospital
◯ Southmead Hospital
◯ Southport and Formby District General Hospital
◯ St Albans City Hospital
◯ St Bartholomews Hospital
◯ St George's Hospital
◯ St Helier Hospital
◯ St James' University Hospital
◯ St Mary's Hospital
◯ St Mary's Hospital
◯ St Peter's Hospital
◯ St Richard's Hospital
◯ St Thomas' Hospital
◯ Stepping Hill Hospital
◯ Stoke Mandeville Hospital
◯ Sunderland Royal Hospital
◯ Sussex Orthopaedic Treatment Centre
◯ Tameside Hospital
◯ The Christie Hospital
◯ The County Hospital, Wye Valley
◯ The James Cook University Hospital
◯ The Royal Liverpool University Hospital
◯ The Royal London Hospital
◯ The Royal Marsden Hospital, Chelsea
◯ The Royal Marsden Hospital, Sutton
◯ The Whittington Hospital
◯ The York Hospital
◯ Torbay Hospital
◯ Trafford General Hospital
◯ Treliske Hospital
◯ University College Hospital
◯ University College Hospital at Westmoreland Street
◯ University Hospital Aintree
◯ University Hospital Coventry
◯ University Hospital Lewisham
◯ University Hospital of Hartlepool
◯ University Hospital of North Durham
◯ University Hospital of North Tees
◯ Walsall Manor Hospital
◯ Wansbeck General Hospital
◯ Warrington Hospital
◯ Warwick Hospital
◯ Watford General Hospital
◯ West Cumberland Hospital
◯ West Middlesex University Hospital
◯ West Suffolk Hospital
◯ Weston General Hospital
◯ Wexham Park Hospital
◯ Whipps Cross University Hospital
◯ Whiston Hospital
◯ William Harvey Hospital
◯ Worcestershire Royal Hospital
◯ Worthing Hospital
◯ Wrightington Hospital
◯ Wycombe Hospital
◯ Wythenshawe Hospital
◯ Yeovil District Hospital

1b. Which Northern Irish hospital site are you completing this form for?

- ◯ Not listed
- ◯ Altnagelvin Hospital
- ◯ Antrim Area Hospital
- ◯ Causeway Hospital
- ◯ Royal Victoria Hospital
- ◯ South West Acute Hospital
- ◯ Ulster Hospital

1b. Which Scottish hospital site are you completing this form for?

- ◯ Not listed
- ◯ Dumfries and Galloway Royal Infirmary
- ◯ Gartnavel General Hospital
- ◯ Gilbert Bain Hospital
- ◯ Glasgow Royal Infirmary
- ◯ Golden Jubilee National Hospital
- ◯ Hairmyres Hospital
- ◯ Institute of Neurological Sciences
- ◯ Inverclyde Royal Hospital
- ◯ Lorn and Islands Hospitall
- ◯ Monklands District General Hospital
- ◯ New Victoria Hospital
- ◯ Ninewells Hospital
- ◯ Perth Royal Infirmary
- ◯ Queen Elizabeth University Hospital
- ◯ Raigmore Hospita
- ◯ Royal Alexandra Hospital
- ◯ Royal Infirmary of Edinburgh
- ◯ St John's Hospital
- ◯ Western General Hospital
- ◯ Wishaw General Hospital
- ◯ University Hospital Ayr
- ◯ University Hospital Crosshouse
- ◯ Victoria Hospital

1b. Which Welsh hospital site are you completing this form for?

- ◯ Not listed
- ◯ Bronglais Hospital
- ◯ Glan Clwyd Hospital
- ◯ Glangwili General Hospital
- ◯ Morriston Hospital
- ◯ Nevill Hall Hospital
- ◯ Prince Charles Hospital
- ◯ Princess of Wales Hospital
- ◯ Royal Glamorgan Hospital
- ◯ Royal Gwent Hospital
- ◯ Singleton Hospital
- ◯ Withybush Hospital
- ◯ Wrexham Maelor Hospital
- ◯ University Hospital of Wales
- ◯ Ysbyty Gwynedd

1c. If your hospital isn't listed above then please type the name of your hospital here.

____

2. How many 'general and acute' beds are there at your hospital?

Please use the NHS data dictionary definition of a bed, in summary:

-Consultant led bed for NHS patient use

-Any device that may be used to permit a patient to lie down when the need to do so is as a consequence of the patient's condition rather than the need for active intervention such as examination, diagnostic investigation, manipulation/treatment, or transport.

-Includes cots for unwell babies eg. special care baby unit cots (not cots for well babies or neonatal intensive care cots)

-Excludes critical care beds (including adult, paediatric and neonatal intensive care beds/cots), private patient beds, beds led by non-consultants (midwifery, therapist or GP led) and obstetrician led beds.

For a full definition please visit https://www.england.nhs.uk/statistics/wp-content/uploads/sites/2/2021/06/KH03-Guidance-June-2021.pdf.

The CQC sometimes record this information in their reports at https://www.cqc.org.uk/what-we-do/how-we-do-our-job/inspection-reports.

____
(If you know an exact answer please provide it, otherwise please answer to the nearest 50.)

3. How would you describe your hospital? Choose the best answer

- ◯ Teaching hospital - Major Trauma Centre
- ◯ Teaching hospital - not a Major Trauma Centre
- ◯ District general hospital
- ◯ Community hospital
- ◯ Treatment centre
- ◯ Independent sector hospital
- ◯ Other

3a. If you answered 'Other' to question 3, then please specify

____

4. Which interventional specialities does your hospital provide? Tick all that apply.	☐ Abdominal: hepatobiliary<br>☐ Abdominal: lower GI<br>☐ Abdominal: upper GI<br>☐ Abdominal: other<br>☐ Burns<br>☐ Cardiac surgery<br>☐ ENT<br>☐ Interventional cardiology<br>☐ Interventional radiology<br>☐ Maxillo-facial and dental<br>☐ Gastroenterology<br>☐ General Surgery<br>☐ Gynaecology<br>☐ Neurosurgery<br>☐ Ophthalmology<br>☐ Orthopaedics - elective<br>☐ Orthopaedics - trauma<br>☐ Pain<br>☐ Plastics<br>☐ Spinal surgery<br>☐ Thoracic surgery<br>☐ Transplant surgery<br>☐ Urology<br>☐ Vascular surgery<br>☐ Other
4a. If you answered 'Other' to question 4, then please specify which other interventional specialities your hospital provides	________________________________
5. Does your hospital have a named lead anaesthetist for perioperative medicine?	◯ Yes<br>◯ No<br>◯ Don't know
6. Does your hospital have a named lead anaesthetist for cognitive impairment?	◯ Yes<br>◯ No<br>◯ Don't know
7. Does your hospital have a named member of staff who is accountable for the care of older people with frailty whilst in hospital (from any speciality)?	◯ Yes<br>◯ No<br>◯ Don't know
8. Does your hospital have guidelines on the prevention and management of delirium?	◯ Yes<br>◯ No<br>◯ Don't know
9. Does your hospital have a pathway for the perioperative management of people living with frailty undergoing surgery?	◯ Yes<br>◯ No<br>◯ Don't know

Preoperative Anaesthetic Assessment

SNAP 3 would like to collect data regarding preoperative assessment during September 2021. This question refers to adult services.

You may need to refer to the following colleagues to answer these questions:

Service managers Assistant general managers/general managers Business objectives teams/software using cons names/clinic codes Informatics/information analysis /health informatics teams Clinic clerks Matrons or specialist nurses

10. Are you able to find out how many patients had a face to face review by nursing staff, in the preoperative anaesthetic assessment clinic during the month of September 2021?

◯ Yes
◯ No, I am unable to find this information
(Include reviews from 1st- 30th September 2021)

10a. How many patients had a face to face review by nursing staff, in the preoperative anaesthetic assessment clinic during the month of September 2021?

____
(Include reviews from 1st- 30th September 2021)

11. Are you able to find out many patients had a telephone review by nursing staff, in the preoperative anaesthetic assessment clinic during the month of September 2021?

◯ Yes
◯ No, I am unable to find this information
(Include reviews from 1st- 30th September 2021)

11a. How many patients had a telephone review by nursing staff, in the preoperative anaesthetic assessment clinic during the month of September 2021?

____
(Include reviews from 1st- 30th September 2021)

12. Are you able to find out how many patients had a face to face review by an anaesthetist, in the preoperative anaesthetic assessment clinic during the month of September 2021?

◯ Yes
◯ No, I am unable to find this information
(Include reviews from 1st- 30th September 2021)

12a. How many patients had a face to face review by an anaesthetist, in the preoperative anaesthetic assessment clinic during the month of September 2021?

____
(Include reviews from 1st- 30th September 2021)

13. Are you able to find out how many patients had a telephone review by an anaesthetist, in the preoperative anaesthetic assessment clinic during the month of September 2021?

◯ Yes
◯ No, I am unable to find this information
(Include reviews from 1st- 30th September 2021)

13a. How many patients had a telephone review by an anaesthetist, in the preoperative anaesthetic assessment clinic during the month of September 2021?

____
(Include reviews from 1st- 30th September 2021)

14. Are you able to find out how many patients had a notes review by an anaesthetist, in the preoperative anaesthetic assessment clinic during the month of September 2021?

◯ Yes
◯ No, I am unable to find this information
(Include reviews from 1st- 30th September 2021)

14a. How many patients had a notes review by an anaesthetist, in the preoperative anaesthetic assessment clinic during the month of September 2021?	______________________ (Include reviews from 1st- 30th September 2021)
15. Please describe any other ways that patients were reviewed by the anaesthetic led preoperative assessment clinic	____________________________
15a. How many patients were reviewed using this additional method?	______________________
16. Are you able to find out how many consultant PAs are currently allocated to anaesthetic preoperative assessment per week?	◯ Yes<br>◯ No, I am unable to find this information
16a. How many consultant PAs are currently allocated to anaesthetic preoperative assessment per week?	______________________

The numbers of patients reviewed and number of consultant PA's dedicated to preoperative assessment are really important to SNAP 3. Often this information is held by hospital managers and administration staff.

We would be really grateful if you could investigate further and come back to the survey at a later date. If you click 'Save & Return Later' at the bottom of the page, then you will be provided with a unique website address in your browser. This can be copied/bookmarked in order to return to your hospital's partially completed survey. There is also an option to have the partially completed survey link emailed to an address of your choice.

Thank you for your support of SNAP 3.

Preoperative Geriatrician Assessment

SNAP 3 would like to collect data regarding preoperative assessment by geriatricians during September 2021.

You may need to refer to the following colleagues to answer these questions:

Service managers Assistant general managers/general managers Business objectives teams/software using cons names/clinic codes Informatics/information analysis /health informatics teams Clinic clerks Matrons or specialist nurses

17. Does your hospital have a preoperative geriatric medicine clinic?	◯ Yes<br>◯ No<br>◯ Don't know
18. Are you able to find out how many patients had a face to face review by nursing staff, in the preoperative geriatric medicine clinic during the month of September 2021?	◯ Yes<br>◯ No, I am unable to find this information<br>(Include reviews from 1st- 30th September 2021)
18a. How many patients had a face to face review by nursing staff, in the preoperative geriatric medicine clinic during the month of September 2021?	____________________________<br>(Include reviews from 1st- 30th September 2021)
19. Are you able to find out how many patients had a telephone review by nursing staff, in the preoperative geriatric medicine assessment clinic during the month of September 2021?	◯ Yes<br>◯ No, I am unable to find this information<br>(Include reviews from 1st- 30th September 2021)
19a. How many patients had a telephone review by nursing staff, in the preoperative geriatric medicine assessment clinic during the month of September 2021?	____________________________<br>(Include reviews from 1st- 30th September 2021)
20. Are you able to find out how many patients had a face to face review by a geriatrician, in the preoperative geriatric medicine assessment clinic during the month of September 2021?	◯ Yes<br>◯ No, I am unable to find this information<br>(Include reviews from 1st- 30th September 2021)
20a. How many patients had a face to face review by a geriatrician, in the preoperative geriatric medicine assessment clinic during the month of September 2021?	____________________________<br>(Include reviews from 1st- 30th September 2021)
21. Are you able to find out how many patients had a telephone review by a geriatrician, in the preoperative geriatric medicine assessment clinic during the month of September 2021?	◯ Yes<br>◯ No, I am unable to find this information<br>(Include reviews from 1st- 30th September 2021)
21a. How many patients had a telephone review by a geriatrician, in the preoperative geriatric medicine assessment clinic during the month of September 2021?	____________________________<br>(Include reviews from 1st- 30th September 2021)

22. Are you able to find out how many patients had a notes review by a geriatrician, in the preoperative geriatric medicine assessment clinic during the month of September 2021?	◯ Yes<br>◯ No, I am unable to find this information<br>(Include reviews from 1st- 30th September 2021)
22a. How many patients had a notes review by a geriatrician, in the preoperative geriatric medicine assessment clinic during the month of September 2021?	__________________________________<br>(Include reviews from 1st- 30th September 2021)
23. Please describe any other ways that patients were reviewed by the preoperative geriatric medicine clinic	____
23a. If you entered an additional method of reviewing patients, approximately how many patients were reviewed in this manner?	__________________________________
24. Are you able to find out how many consultant PAs are currently allocated to geriatric medicine preoperative assessment per week?	◯ Yes<br>◯ No, I am unable to find this information
24a. How many consultant PAs are currently allocated to geriatric medicine preoperative assessment per week?	__________________________________

The numbers of patients reviewed and consultant PA's dedicated to preoperative assessment are really important to SNAP 3. Often this information is held by hospital managers and administration staff.

We would be really grateful if you could investigate further and come back to the survey at a later date. If you click 'Save & Return Later' at the bottom of the page, then you will be provided with a unique website address in your browser. This can be copied/bookmarked in order to return to your hospital's partially completed survey. There is also an option to have the partially completed survey link emailed to an address of your choice.

Thank you for your support of SNAP 3.

26. How are preoperative clinician-to-clinician discussions about high-risk older patients conducted? Tick all that apply	☐ MDT formal meeting (face to face or virtual meeting with the appropriate staff relevant to an individual patient's circumstances)<br>☐ Ad hoc emails, telephone calls or conversations<br>☐ Ad hoc joint patient consultations with anaesthetists and surgeons<br>☐ Ad hoc joint patient consultations with anaesthetists and geriatricians<br>☐ Ad hoc joint patient consultations with geriatricians and surgeons<br>☐ Joint clinics between anaesthetists and surgeons<br>☐ Joint clinics between anaesthetists and geriatricians<br>☐ Joint clinics between geriatricians and surgeons<br>☐ Other, please specify<br>☐ Don't know
26a. If you answered 'Other' to question 26 then please specify how preoperative clinician-to-clinician discussions are conducted	____

Perioperative Frailty Assessment

27. Is frailty assessment routinely documented for older patients, in the following settings? Please select all answers that apply.
This question refers to patients who are at least 60 years old.

	For all patients 60 years+	For selected patients	Rarely or inconsistently	Never	N/A	Don't know
At nurse led anaesthetic preoperative assessment clinic	◯	◯	◯	◯	◯	◯
At anaesthetist led anaesthetic preoperative assessment clinic	◯	◯	◯	◯	◯	◯
At geriatric medicine preoperative assessment clinic	◯	◯	◯	◯	◯	◯
During admission for elective surgery	◯	◯	◯	◯	◯	◯
During admission for emergency surgery	◯	◯	◯	◯	◯	◯

27a. If preoperative frailty assessment is documented as occuring anywhere else, please specify where this occurs eg. surgical clinic

____

28. What triggers frailty assessment in elective surgical patients? Tick all that apply

- ☐ Routine based on age criteria (specify age)
- ☐ Routine based on type of operation
- ☐ Routine based on speciality eg. Triggered by spinal, orthopaedic, vascular surgery
- ☐ Clinician judgement
- ☐ Other, please specify
- ☐ Don't know
- ☐ N/A

28a. What are the age criteria for assessment of frailty in elective surgical patients?

- ◯ 65 years or older
- ◯ 70 years or older
- ◯ 75 years or older
- ◯ 80 years or older
- ◯ 85 years or older
- ◯ Other, please specify

28b. What is the age criteria for assessment of frailty in elective surgical patients?

____

28c. What else triggers the assessment of frailty in elective surgical patients?

____

29. What triggers frailty assessment in emergency patients? Tick all that apply

- ☐ Routine based on age criteria (specify age)
- ☐ Routine based on type of operation
- ☐ Routine based on speciality eg. Triggered by spinal, orthopaedic, vascular surgery
- ☐ Clinician judgement
- ☐ Other, please specify
- ☐ N/A
- ☐ Don't know

29a. What are the age criteria for assessment of frailty in emergency surgical patients?	◯ 65 years or older<br>◯ 70 years or older<br>◯ 75 years or older<br>◯ 80 years or older<br>◯ 85 years or older<br>◯ Other, please specify
29b. what is the age criteria for assessment of frailty in emergency surgical patients?	__________________________
29c. What else triggers the assessment of frailty in emergency surgical patients?	__________________________
30. Which frailty assessment tools are routinely used in your hospital? Tick all that apply	☐ Clinical Frailty Scale/Rockwood Frailty Scale<br>☐ Edmonton Frailty Scale<br>☐ Groningen Frailty Indicator<br>☐ Gait Speed Test<br>☐ PRISMA-7<br>☐ Risk Analysis Index-C<br>☐ Timed Up and Go (TUG) Test<br>☐ Electronic Frailty Index<br>☐ Hospital Risk Frailty Index<br>☐ Grip Strength<br>☐ Comprehensive Geriatric Assessment<br>☐ Clinician judgement/discretion<br>☐ Other, please specify<br>☐ Don't know
30a. If you answered 'Other' to question 30 then please specify which other tools are used to assess frailty	__________________________

Perioperative Cognitive Assessment

31. Is a cognitive assessment routinely documented for older patients, in the following settings?
Please tick all answers that apply.
This question refers to patients who are at least 60 years old

	For all patients 60 years+	For selected patients	Rarely or inconsistently	Never	N/A	Don't know
At nurse led anaesthetic preoperative assessment clinic	◯	◯	◯	◯	◯	◯
At anaesthetist led anaesthetic preoperative assessment clinic	◯	◯	◯	◯	◯	◯
At geriatric medicine preoperative assessment clinic	◯	◯	◯	◯	◯	◯
During admission for elective surgery	◯	◯	◯	◯	◯	◯
During admission for emergency surgery	◯	◯	◯	◯	◯	◯

31a. If cognitive assessment is documented as occuring anywhere else, please specify where this occurs eg. surgical clinic

____

32. What triggers the assessment of cognition in the elective surgical setting? Tick all that apply

- ☐ Routine based on age criteria (specify which)
- ☐ Routine based on type of operation
- ☐ Routine based on speciality eg. Triggered by spinal, orthopaedic, vascular surgery
- ☐ Clinician judgement
- ☐ Other, please specify
- ☐ Don't know
- ☐ N/A

32a. Which age criteria triggers cognitive assessment in elective surgical patients?

- ◯ 65 years or older
- ◯ 70 years or older
- ◯ 75 years or older
- ◯ 80 years or older
- ◯ 85 years or older
- ◯ Other, please specify

32b. What is the age criteria that triggers cognitive assessment in elective surgical patients?

____

32c. If you answered 'Other' to question 32 , please specify what triggers the assessment of cognition in elective surgical patients

____

33. What triggers the assessment of cognition in the emergency setting? Tick all that apply	☐ Routine based on age criteria (specify which)<br>☐ Routine based on type of operation<br>☐ Routine based on speciality eg. Triggered by spinal, orthopaedic, vascular surgery<br>☐ Clinician judgement<br>☐ Other, please specify<br>☐ Don't know<br>☐ N/A
33a. Which age criteria triggers cognitive assessment in emergency surgical patients?	○ 65 years or older<br>○ 70 years or older<br>○ 75 years or older<br>○ 80 years or older<br>○ 85 years or older<br>○ Other, please specify
33b. What is the age criteria that triggers cognitive assessment in emergency surgical patients?	____________________________
33c. If you answered 'Other' to question 33 , please specify what triggers the assessment of cognition in the emergency setting	__________________________________
34. Which cognition assessment tools are used to assess surgical patients? Tick all that apply	☐ Single dementia question screen<br>☐ Mini mental state examination (MMSE)<br>☐ 4AT<br>☐ Abbreviated mental test (AMT)<br>☐ Montreal Cognitive Assessment (MOCA)<br>☐ Mini-Cog<br>☐ AD8 Dementia Screening<br>☐ 10-point Cognitive Screener (10-CS)<br>☐ 6-item Cognitive Impairment Test (6CIT)<br>☐ 6-item Screener<br>☐ Memory Impairment Screen (MIS)<br>☐ Other, please specify<br>☐ None<br>☐ Don't know
34a. If you answered 'other' to question 33 then please specify which cognition assessment tool is used	____________________________

Risk Assessment for Postoperative Delirium

35. Is a risk assessment for postoperative delirium routinely documented for older patients in the following settings?
Please choose all answers that apply.
This question refers to patients who are at least 60 years old.

	For every patient 60 years+	For selected patients	Rarely or inconsistently	Never	N/A	Don't know
At nurse led anaesthetic preoperative assessment clinic	○	○	○	○	○	○
At anaesthetist led anaesthetic preoperative assessment clinic	○	○	○	○	○	○
At geriatric medicine preoperative assessment clinic	○	○	○	○	○	○
During admission for elective surgery	○	○	○	○	○	○
During admission for emergency surgery	○	○	○	○	○	○

35a. If risk of postoperative delirium is documented as occuring anywhere else, please specify where this occurs eg. surgical clinic

____

36. What triggers the risk assessment for postoperative delirium in elective surgical patients? Tick all that apply

- ☐ Routine based on age criteria (specify which)
- ☐ Routine based on type of operation
- ☐ Routine based on speciality eg. Triggered by spinal, orthopaedic, vascular surgery
- ☐ Clinician judgement
- ☐ Other, please specify
- ☐ Don't know

36a. Which age criteria triggers assessment of postoperative delirium risk in elective patients?

- ○ 65 years or older
- ○ 70 years or older
- ○ 75 years or older
- ○ 80 years or older
- ○ 85 years or older
- ○ Other, please specify

36b. What is the age criteria that triggers assessment of risk for postoperative delirium in elective patients?

____

36c. If you answered 'Other' to question 25, then please specify what triggers a risk assessment for postoperative delirium in elective patients

____

37. What triggers the risk assessment for postoperative delirium in emergency surgical patients? Tick all that apply	☐ Routine based on age criteria (specify which)<br>☐ Routine based on type of operation<br>☐ Routine based on speciality eg. Triggered by spinal, orthopaedic, vascular surgery<br>☐ Clinician judgement<br>☐ Other, please specify<br>☐ Don't know
37a. What is the age criteria that triggers assessment of risk for postoperative delirium in emergency surgical patients?	◯ 65 years or older<br>◯ 70 years or older<br>◯ 75 years or older<br>◯ 80 years or older<br>◯ 85 years or older<br>◯ Other, please specify
37b. What is the age criteria that triggers assessment of risk for postoperative delirium in emergency surgical patients?	__________________________________
37c. If you answered 'Other' to question 26, then please specify what triggers a risk assessment for postoperative delirium in emergency surgical patients	____

38. How are older surgical patients assessed for their risk of developing postoperative delirium?

- ☐ Clinical judgement
- ☐ Validated delirium risk assessment tool eg. Delirium Elderly At- Risk (DEAR) instrument, Inouye et al, please specify
- ☐ Locally adapted delirium assessment tool, please describe
- ☐ Other, please specify
- ☐ Don't know

(Inouye, S. K., Viscoli, C. M., Horwitz, R. I., Hurst, L. D., & Tinetti, M. E. (1993). A predictive model for delirium in hospitalized elderly medical patients based on admission characteristics. Annals of internal medicine, 119(6), 474-481. https://doi.org/10.7326/0003-4819-119-6-199309150-0000[illegible]

38a. If you answered 'other' to question 38, how else are older surgical patients assessed for their risk of developing postoperative delirium?

____

38b. Which validated risk prediction tool for delirium does your hospital use?

____
(eg. Inouye et al, Delphi score, DEAR instrument)

38c. Please describe the locally adapted delirium assessment tool that your hospital uses

____

39. Which tools are routinely used to identify delirium in your hospital? Tick all that apply

- ☐ 4AT
- ☐ Confusion Assessment Method (CAM)
- ☐ Single question identifying delirium (SQiD)
- ☐ Abbreviated Mental Test Score (AMTS)
- ☐ Months of the year backwards or serial sevens
- ☐ Recognising acute delirium as part of your routine (RADAR)
- ☐ Nursing-DElirium SCore (Nu-DESC)
- ☐ Delirium Observational Screening Scale (DOSS)
- ☐ Modified Richmond Agitation and Sedation Scale (mRASS)
- ☐ Delirium rating scale (DRS-98R)
- ☐ Delirium symptom interview
- ☐ Memorial Delirium Assessment Scale (MDAS)
- ☐ Organic Brain Syndrome Scale
- ☐ DSM-V (Diagnostic and Statistical Manual of Mental Disorders, 5th edition)
- ☐ Spatial span forwards
- ☐ None of the above
- ☐ Other, please specify
- ☐ Don't know

39a. If you answered 'other' to question 39, how else are patients assessed for delirium?

____

Postoperative Care

40. Are you able to find out how many dedicated consultant PAs are currently allocated to anaesthetic delivered postoperative care for surgical patients (for instance, as part of a perioperative medicine team, or similar) per week?

Note: Exclude PAs allocated for post-acute care units [PACU], overnight intensive care [OIR], enhanced care, critical care, and pain services. Do not include any PA allocation where consultants are given time to review their own patients before or after their sessions in theatre.

◯ Yes
◯ No, I am unable to find this information

40a. How many dedicated consultant PAs are currently allocated to anaesthetic delivered postoperative care for surgical patients (for instance, as part of a perioperative medicine team, or similar) per week?

Note: Exclude PAs allocated for post-acute care units [PACU], overnight intensive care [OIR], enhanced care, critical care, and pain services. Do not include any PA allocation where consultants are given time to review their own patients before or after their sessions in theatre.

____

41. Can you find out how many consultant PAs are currently allocated to geriatric medicine delivered postoperative care for surgical patients per week?

◯ Yes
◯ No, I am unable to find this information

41a. How many consultant PAs are currently allocated to geriatric medicine delivered postoperative care for surgical patients per week?

____

The numbers of consultant PA's dedicated to postoperative care are really important to SNAP 3. Often this information is held by hospital managers and administration staff.

We would be really grateful if you could investigate further and come back to the survey at a later date. If you click 'Save & Return Later' at the bottom of the page, then you will be provided with a unique website address in your browser. This can be copied/bookmarked in order to return to your hospital's partially completed survey. There is also an option to have the partially completed survey link emailed to an address of your choice.

Thank you for your support of SNAP 3.

42. How is postoperative care for older high-risk patients who have a hip fracture provided? Tick any that apply.

Please exclude post-acute care units, overnight intensive care, enhanced care, critical care from your answer.

	Scheduled, proactive, ward round or consult	Acute, referral required, reactive consult	Multidisciplinary team meeting (MDM)	None of these	N/A	Don't know
Geriatricians (consultant led team)	☐	☐	☐	☐	☐	☐
Anaesthetists (consultant led team, excluding specialist pain teams)	☐	☐	☐	☐	☐	☐
Intensivists (consultant led team, excluding critical care outreach)	☐	☐	☐	☐	☐	☐
Ward based physicians (consultant led team, who aren't geriatricians)	☐	☐	☐	☐	☐	☐
Clinical nurse specialist responsible for coordinating a patient's care	☐	☐	☐	☐	☐	☐
Allied healthcare professional responsible for coordinating a patient's care	☐	☐	☐	☐	☐	☐

43. How is postoperative care for older high-risk patients who have an elective orthopaedic operation provided? Tick any that apply.

Please exclude post-acute care units, overnight intensive care, enhanced care, critical care from your answer.

	Scheduled, proactive, ward round or consult	Acute, referral required, reactive consult	Multidisciplinary team meeting (MDM)	None of these	N/A	Don't know
Geriatricians (consultant led team)	☐	☐	☐	☐	☐	☐
Anaesthetists (consultant led team, excluding specialist pain teams)	☐	☐	☐	☐	☐	☐
Intensivists (consultant led team, excluding critical care outreach)	☐	☐	☐	☐	☐	☐
Ward based physicians (consultant led team, who aren't geriatricians)	☐	☐	☐	☐	☐	☐
Clinical nurse specialist responsible for coordinating a patient's care	☐	☐	☐	☐	☐	☐
Allied healthcare professional responsible for coordinating a patient's care	☐	☐	☐	☐	☐	☐

44. How is postoperative care for older high-risk patients who have an emergency laparotomy provided? Tick any that apply.

Please exclude post-acute care units, overnight intensive care, enhanced care, critical care from your answer.

	Scheduled, proactive, ward round or consult	Acute, referral required, reactive consult	Multidisciplinary team meeting (MDM)	None of these	N/A	Don't know
Geriatricians (consultant led team)	☐	☐	☐	☐	☐	☐
Anaesthetists (consultant led team, excluding specialist pain teams)	☐	☐	☐	☐	☐	☐
Intensivists (consultant led team, excluding critical care outreach)	☐	☐	☐	☐	☐	☐
Ward based physicians (consultant led team, who aren't geriatricians)	☐	☐	☐	☐	☐	☐
Clinical nurse specialist responsible for coordinating a patient's care	☐	☐	☐	☐	☐	☐
Allied healthcare professional responsible for coordinating a patient's care	☐	☐	☐	☐	☐	☐

45. How is postoperative care for older high-risk patients who have elective general surgery provided? Tick any that apply

Please exclude post-acute care units, overnight intensive care, enhanced care, critical care from your answer.

	Scheduled, proactive, ward round or consult	Acute, referral required, reactive consult	Multidisciplinary team meeting (MDM)	None of these	N/A	Don't know
Geriatricians (consultant led team)	☐	☐	☐	☐	☐	☐
Anaesthetists (consultant led team, excluding specialist pain teams)	☐	☐	☐	☐	☐	☐
Intensivists (consultant led team, excluding critical care outreach)	☐	☐	☐	☐	☐	☐
Ward based physicians (consultant led team, who aren't geriatricians)	☐	☐	☐	☐	☐	☐
Clinical nurse specialist responsible for coordinating a patient's care	☐	☐	☐	☐	☐	☐
Allied healthcare professional responsible for coordinating a patient's care	☐	☐	☐	☐	☐	☐

Thank you!

We are really grateful to you for completing this survey.

If you are unable to answer all the questions then please discuss with colleagues in perioperative medicine, preoperative assessment or surgery. By clicking on 'Save & Return Later', you can save your answers and access a web address that can be passed onto other colleagues to allow them to update answers as necessary.

If you have any comments that you'd like to share with SNAP 3 regarding perioperative care services, then please use the comment box.

If you would like to be named in the collaborator list for SNAP 3, then please ensure your site's Principal Investigator has your details.

____

Please enter your email address if you are happy to be contacted with any queries about your hospital's survey response.

____
(This email address will not be distributed or used for any other purposes. It will be deleted once data has been analysed.)
